# Toward the upscaling of school nutrition programs in Dubai: An exploratory study

**DOI:** 10.3389/fpubh.2022.1038726

**Published:** 2022-11-07

**Authors:** Reem AlGurg, Nour Abu Mahfouz, Farah Otaki, Mohamad Alameddine

**Affiliations:** ^1^College of Medicine, Mohammed Bin Rashid University of Medicine and Health Sciences, Dubai, United Arab Emirates; ^2^Strategy and Institutional Excellence, Mohammed Bin Rashid University of Medicine and Health Sciences (MBRU), Dubai, United Arab Emirates; ^3^College of Health Sciences, University of Sharjah, Sharjah, United Arab Emirates

**Keywords:** school nutrition programs, school-aged children, nutrition, Dubai, United Arab Emirates (UAE), key informant interviews, health systems, thematic analysis

## Abstract

**Background:**

School nutrition programs impact the intellectual, social, and emotional development of school children, as well as their future risk of developing Non-Communicable Diseases. While many stakeholders are involved in the development, implementation, and evaluation of school nutrition programs in Dubai, United Arab Emirates, little is known about the complementarity among those stakeholders, and the means to upscale school nutrition programs while ensuring effective, efficient, and equitable implementation. Accordingly, this study aims at exploring the perceptions of a diverse group of stakeholders, positioned at differing levels of the public health and education ecosystems in the United Arab Emirates, in relation to current guidelines and practices around the planning, implementation, and evaluation of school nutrition programs in Dubai, United Arab Emirates.

**Methods:**

The current study relied on a qualitative design, based on semi-structured key informant interviews. A total of 29 interviews were carried out. Those interviewees included leaders and directors from different institutions, decision- and policy- makers, nutritionists and dieticians, school nurses and nurse managers, and school principals and vice principals. All stakeholders were interviewed by the research team. Data was transcribed, and then thematically analyzed using the health systems' model as an analytic framework.

**Results:**

The thematic analysis of interview data identified five interrelated themes. The first theme relates to the limited coordination across regulatory local and federal entities, and the multiplicity of guidelines issued by the different stakeholders. The challenges around the human and financial resourcing of school nutrition programs constituted the second theme. The third theme was the weakly coordinated implementation efforts. The fourth theme was the need for better performance measurement, and the fifth theme flagged the need for improved inclusiveness for health needs and cultural preferences of the diverse student body in Dubai (given that there are citizens from more than 200 nationalities co-existing in Dubai).

**Conclusion:**

This study emphasizes that all the involved stakeholders need to better collaborate to upscale the school nutrition program in Dubai. This will require the formation of a unified governing body, which would identify and develop a single stream of resources, and sets in place a reliable, all encapsulating and equitable implementation plan along with an overarching monitoring and evaluation framework.

## Background

Several international nutrition-based organizations strongly recommend that schools adapt reliable and comprehensive nutritional program, and promote nutrition education, as well as strengthen school-home-community partnerships (1), to maximize the positive impact on the general health of a community (2, 3). This is due to the significant impact of School Nutrition Programs (SNPs) on the eating behaviors, and health and wellbeing of school children (4–6). SNPs could also impact the intellectual, social, and emotional development of school student, as well as their cognitive function and academic performance (7, 8). The effects of SNPs are not limited to the attitudes and behaviors of students in the school setting, since they could positively influence eating habits at home by enhancing mother's knowledge on health, nutrition, and childcare (1, 9–11). Furthermore, SNPs are proven to decrease the school children's risk for Non-Communicable Diseases (12). However, all the above-mentioned positive effects of SNPs hinge on the effective planning for and implementation of those programs (13). Hence, for any country to reap the benefits of SNPs, it is imperative to develop a better understanding of the programs in place, as well as the enablers and barriers for their proper implementation (3, 14). This is of pivotal importance in countries where school children overweight and obesity are epidemic.

To the best of the researchers' knowledge, no study has been conducted to capture such information in relation to SNPs in the United Arab Emirates (UAE). Accordingly, this study aims at exploring the perception of key stakeholders, positioned at differing levels of the public health and education ecosystems in the UAE, in relation to SNPs in Dubai and the main opportunities for improvement. As such, the current study offers a thorough understanding of the status-quo of the SNPs in Dubai, and in turn evidence-driven suggestions for improvement and development.

## Methods

### Context

This study took place at Dubai, UAE. With close to three million (a third of the country's population) inhabitants, Dubai is the most populated Emirate of the UAE (15). In the Academic Year 2018–2019, Dubai reported 280,979 registered students in private schools and 29,387 students in public ones (15).

In 2017 and 2018, the prevalence of obesity among UAE in-school children was 13.7 and 13.4%, respectively (16). The target of the national agenda indicator was to reduce this value to 12% by the year 2021 (16). Unfortunately, in 2020, the respective value was raised to around 17%, which can be attributed to the lifestyle restrictions that accompanied Coronavirus Disease of 2019 (COVID-19) (16). Moreover, although there appears to be, since then, plenty of initiatives around improving the quality-of-life of the citizens of the UAE, including but not limited to in-school children (17) (to the best of the authors' knowledge), there are no recent updates on the value of the respective indicator.

Multiple organizations and authorities in the country are working to raise the value of this indicator including: the Ministry of Health and Prevention (MOHAP), Department of Health- Abu Dhabi, Dubai Health Authority (DHA), Dubai Municipality, and the Ministry of Education (MOE). While each of these entities has its own programs, activities, and strategies, they all acknowledge that the reduction of school children obesity, and the enhancement of their health and wellbeing would not be optimally realized without the proper implementation of a nationally endorsed SNP. Anecdotal evidence reveals inconsistencies in the implementation of SNPs in Dubai schools with no clear information on the enablers and barriers to such implementation.

The UAE has several school nutrition guidelines generated by differing federal and Emirate level governing bodies, including the MOE, DHA, Dubai Municipality, MOHAP, and the Health Department-Abu Dhabi (18). The UAE does not have a national policy specific for school nutrition, but rather a national Nutrition Strategy that has a few subsections related to schools (19). Although all the entities are working to reduce the prevalence of childhood obesity, there remain major discrepancies across the guidelines that are affecting the planning, implementation, and monitoring and evaluations of SNPs. The various guidelines and requirements cover differing aspects of food and nutrition in schools in Dubai. These run from pinpointing general requirements, guidelines to prepare meals and snacks, and lists of foods restricted in schools, all the way to defining training requirements of employees and school management, and the roles and responsibilities for schools in promoting health eating. Although food and nutrition education is frequently alluded to in the corresponding documents, there appears to be no unified clearly articulated education strategy is set in place (18). In Dubai, public schools are following the MOE guidelines, while private schools are following the DHA and Dubai Municipality guidelines, along with the Knowledge and Human Development Authority of Dubai (KHDA) recommendations. Public schools end-up having similar programs as they are centrally managed by the MOE, while plans in private schools are quite varied, and depend on the curriculum that the respective school offers.

School canteen suppliers must be approved by the MOE and the Dubai Municipality. For the public schools in Dubai, the MOE is responsible of deciding upon the food suppliers. The role of the school is only supervision, where they only control for the food safety. Private schools, however, have the freedom to choose their suppliers and then get the approval from Dubai Municipality. At the time of this study, several prominent initiatives, in relation to SNPs, were taking place (Table 1).

**Table 1:** Program-based School Nutrition Programs initiatives that were taking place at the time of the study, identified through desk research (i.e., surfing through the webpages of the official websites of the corresponding entities).

**Program**	**Description**	**Responsible authority**	**Level of influence**
School canteen standards and guidelines	A guiding manual for schools to improve the nutritional standard of food served or sold in their premises	Dubai Municipality and Dubai Health Authority	This policy needs to be signed by the school administration and permanently displayed in school canteens
Food labeling	Guidelines to ensure that children have access to nutritious, safe, and wholesome food during their school time	Food Safety Department of Dubai Municipality (Started in 2017)	All schools in Dubai
Happy Schools Initiative	An award to recognize schools that have made achievements in supporting the health and wellbeing of students	Knowledge and Human Development Authority of Dubai (Started in 2017)	Focusing on the wellbeing of Dubai private schools' students between the ages of 10 and 14 years old
Health Awareness Campaigns	Periodic campaigns to boost awareness about healthy eating among students	Ministry of Health and Prevention	Public schools and corresponding community
Guidelines and requirements for food and nutrition in Dubai	Raise awareness of students about the nutrient quotient in foods	Food safety department- Dubai Municipality	Provides all stakeholder with an overview of the health status, nutrition information, nature and effects of foods provided in schools, and common set of nutritional guidelines that would help promote healthy eating in schools in the Emirate of Dubai

### Research design

The study relied on a qualitative design, based on semi-structured key informant interviews. The design enabled the development of an in-depth insight into the perspective of the participants (20). Ethical approval for the study was obtained from the International Review Board (IRB) at the Mohammed Bin Rashid University of Medicine and Health Sciences (MBRU), Dubai, UAE, under the reference number MBRU-IRB-2018-029.

The research team identified key stakeholders who are well-informed and experienced with the SNPs in Dubai. A snowball sampling technique was utilized to recruit additional key informants. Those included leaders and directors from different institutions with clinical and/or management backgrounds, decision- and policymakers, nutritionists and dieticians, nurses and nurse managers, and schools' principals and vice principals. The interviews were continued until the research team attained saturation, where no new concepts were identified (21).

**Figure 1:**
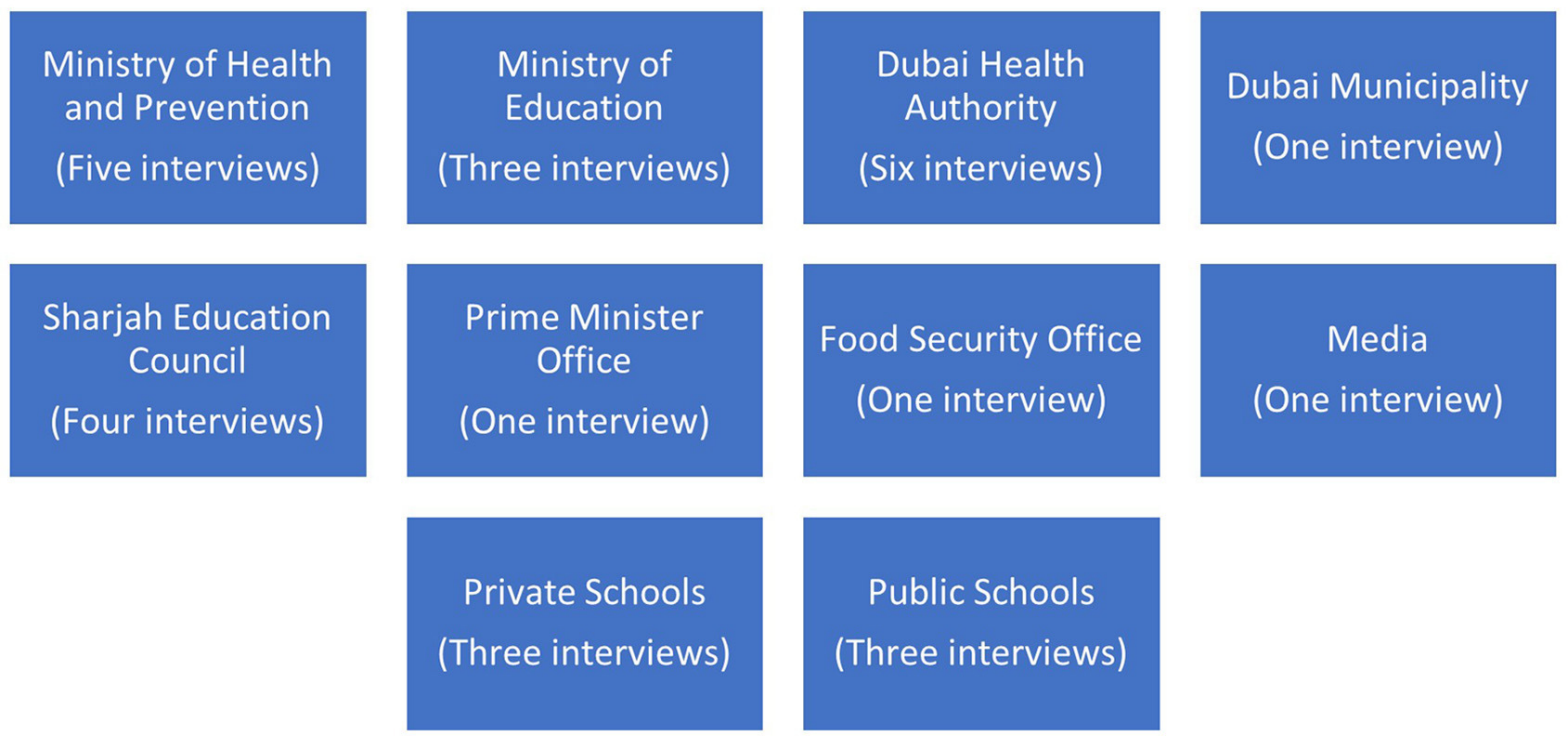
Illustration of the institutions, involved directly or indirectly in SNPs in Dubai.

Out of 48 key informants who were initially recruited, 29 agreed to participate. Those that did not agree to participate attributed this to their busy schedules. When key stakeholders, representing entities, did not agree to participate, they were replaced by other stakeholders from the same entities. Accordingly, semi-structured interviews were conducted with stakeholders from several institutions (Figure 1).

The head of each department, or the person in charge from each institution, received an invitation letter as an attachment to a personalized email. In case of non-response, the team sent an email reminder after 2 weeks of the invitation. If there was no response, the second and final reminder was through a phone call. All interviewees who were interested eventually replied to the email. After that, a mutually convenient interview timing was set with each key informant, and the interviews were conducted accordingly. One day before the interview, a reminder email, with the consent form and participant information sheet, was sent to each of the interviewees. The school stakeholders were invited with the intention to diversify schools by type of curriculum for private schools (British, Indian, or American), and the type of school in terms of the gender of the students for public ones (Male only, Female only or mixed gender).

### Data collection

Semi-structured interviews were conducted from January 14, 2019 through April 18, 2019. The interviews focused on common topics, but with the freedom to pursue additional topics of interest as they surfaced. The interviews were anonymous, but the positions of the interviewees were noted. Before the interviews were conducted, a written informed consent in English, which was also translated into Arabic language, was obtained from all the participants. They were informed about the scope of the study and the overall subject of the questions. The interviewees were informed that they could skip any question they do not wish to answer or terminate the interview at any point.

The interview schedule gathered some basic information on the portfolio of the participants and included the following questions:

1. Please describe your involvement with school nutrition programs in Dubai
2. Please describe any nutrition plans, policies, or programs in the schools you know of or are engaged with,
3. In your professional opinion, how well are the SNPs implemented and evaluated in Dubai?
4. What do you believe are the elements of strength of the SNPs in Dubai?
5. What do you recommend for improving the planning and implementation of SNPs in Dubai? and
6. Do you wish to comment on any other areas related to SNPs in Dubai?
7. Are there any documents you recommend we examine to further understand the SNPs in Dubai?
8. Are their other experts you recommend that we speak with?

Based on the preference of the participant, interviews were carried out in English or Arabic, and were recorded with written consent of the participants. Interviews, lasted on average 45 min, were recorded, transcribed, and (if need be) translated.

### Data analysis

First, the 29 audio-recorded interviews were transcribed verbatim. Interviews in Arabic were translated into English by a member of the research team. A unique alphanumeric identifier was assigned to each participant. This identifier was composed of a unique number for each interviewee, followed by a reference to the level of influence based on the positioning of the corresponding institution (S: School level, ES: Education Sector, E: Emirate, and N: National level), and a reference to the education and development background of the interviewee (C: Clinical, M: Management, and B: Both clinical and management).

Data collected from the interviews were analyzed to identify main themes using the following five-step approach: 1- familiarization, 2- identification of thematic framework, 3- indexing of the transcripts, 4- abstraction and synthesis through charting, and 5- conceptual mapping and interpretation (22, 23).

One member of the research team started by identifying prominent themes from the transcribed interviews which were cross-checked by a second member of the team. In case of disagreement on a theme, discussions took place until the two members reached an agreement. In case an agreement was not reachable after discussion, a third member of the research team provided independent opinion and resolved the disagreement.

Relevant quotes were selected from the individual transcripts and placed under the appropriate themes and sub-themes. All team members reviewed and approved the final themes and sub-themes emanating from the data analysis phase.

## Results

**Figure 2:**
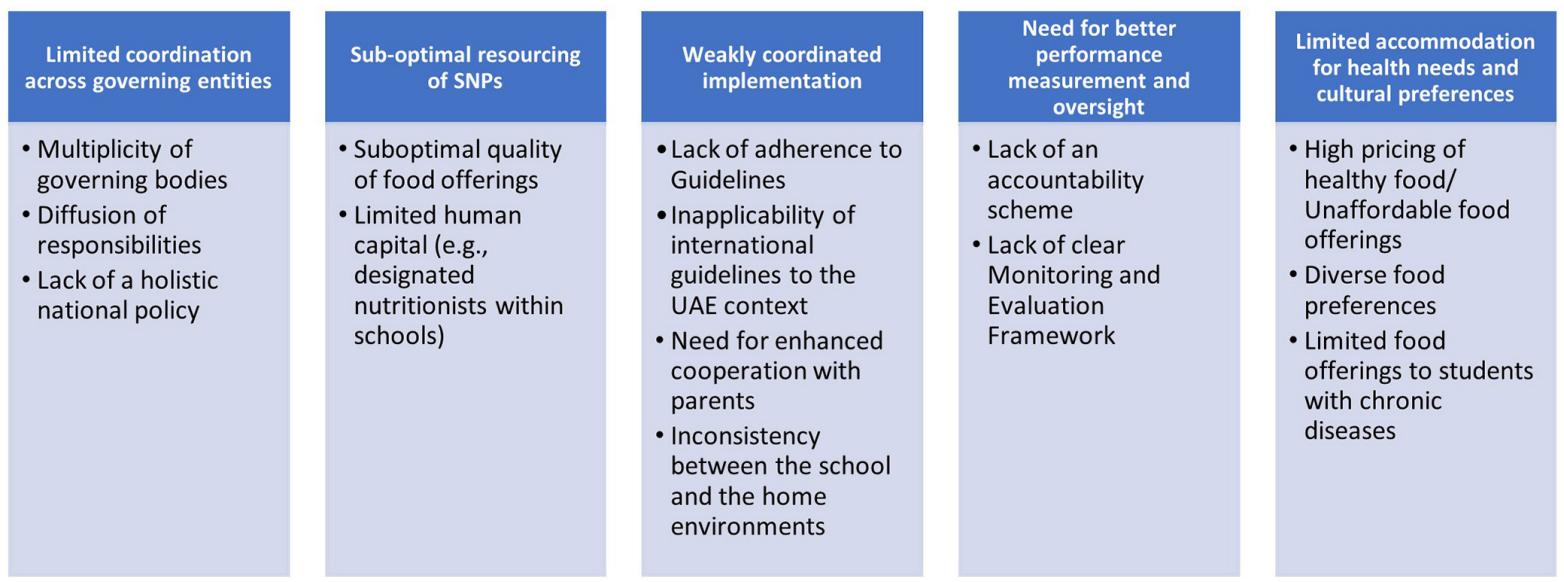
Themes and subthemes generated from this study's qualitative analysis.

The conceptual framework resulting from this study comprised of five key themes, encapsulating 14 sub-themes, summarized in Figure 2.

### Theme 1: Limited coordination across governing entities

Among the most prominent challenges, within the Governance theme, is the multiplicity of regulating bodies, where schools in Dubai need to abide by multiple guidelines of SNPs, issued by different national governing bodies. This is leading to a lot of unnecessary confusion.

5EC: “…the problem is that there are four guidelines for the school canteens. The Ministry of Education has its guidelines. The Dubai Health Authority has another one. There are also guidelines developed and implemented by the Dubai Municipality. The Abu Dhabi Health Authority also has its own guidelines…basically, in the UAE there is no unified policy when it comes to SNPs…”

Other challenges, within this theme, are linked to the apparent diffusion of responsibilities in between the various relevant stakeholders. A manager who is concerned with the health and wellbeing of students in private schools said:

21ESM: “…it is not part of our mandate. They have to take action if they find any violation in schools. The Dubai Municipality needs to take action on that front since they are the entity overseeing the supplying catering companies…”

The same stakeholder highlights that the generated misunderstandings, around roles and responsibilities, are leading to conflicts in between the relevant stakeholders:

21ESM: “…at some stage, we used to be engaged in the process of monitoring the performance of school canteens, but unfortunately, three to four years back, the Ministry of Education sidelined us…”

Another participant expressed that requirements around SNPs are not sufficiently explained in the national framework:

23NM: “…we analyzed the national framework which is more than 100 pages. The school nutrition aspect is touched upon briefly…”

Moreover, another dietician who works for a governmental entity, brought-up the fact that there are no polices for school nutrition at the national level. There is nothing that requires the commitment of the schools in applying the standards.

11ESC: “…currently I do not think that there is a policy or law, and most of the practices are mainly decided by the school itself or the ministry of education…”

### Theme 2: Sub-optimal resourcing of SNPs

A nutritionist working in a federal governing body indicated that high prices of healthy food are a challenge not only at the school level but also at the Emirate level, where the companies supplying the school canteens are profit oriented.

19EC: “…some suppliers will refuse to collaborate with the Ministry of Education, because providing healthy food at regular prices will result in financial loss at their end…”

Moreover, according to a public-school registered nurse, the administration of the SNPs is usually assigned to the existent school nurse. This increases the school nurse workload, and when other priorities are pressing, the SNPs responsibility ends-up falling through the cracks:

15ESC: “…of course, it is not enough to have only the school nurse to handle all health-related aspects because we have huge numbers of students. I already have a lot of important matters to follow-up on. I do not have the space to follow-up with the students who really need help in relation to eating attitudes and behaviors, or to conduct awareness campaigns and in turn evaluate their impact on the respective students. I am really busy; all I end-up doing in relation to SNPs is giving didactic lectures about healthy food options. That is it…”

Similarly, another national policymaker highlighted the importance of having qualified personnel to sustainably implement guidelines:

18NM: “…even when you have the framework or the policy, you still need people to implement it…”

### Theme 3: Weakly coordinated implementation

Apparently, according to a school nurse, raising awareness about healthy behaviors including but not limited to nutrition and dietetics, is not mandated on students. Conducting nutrition-related initiatives, every now and then, is insufficient.

25NM: “…the nutrition program is optional. This in of itself is a problem: why would the students come to the nutrition-related awareness session if it is not mandatory?…”

These discrepancies increase with the schools' lack of adherence to guidelines, where the same informant highlighted:

11ESC: “…since the guidelines are not mandatory, they are not implemented by all schools. It is up to the school's principal and administration to decide whether, or not, to implement the guidelines…”

In addition, another manager, who is involved in overseeing the health and wellbeing of students in private schools, indicated that many of the private schools seem to be operating on an independent basis:

21ESM: “…we give them the tools, and we bring speakers and provide open learning opportunities, but then whether, or not, they put these resources into use is totally up to them…”

Along the same lines, school food quality control officer, working for a regulatory body, mentioned:

12ESC: “…some private schools have their own guidelines. Nothing requires them to keep us (regulators) in the loop…”

When it comes to setting the guidelines, it is usually derived from international standards. A manager at a federal governing body indicated that these benchmarks are not necessarily applicable to the UAE:

2EM: “…the new food portion control system is based on international school nutrition guidelines…some apply British guidelines for food canteens- they are the most applicable, in my opinion. The Canadian and Australian guidelines are not bad, either. I personally do not believe that the American guidelines suit this side of the world- the ideal portion sizes are huge…”

Another senior dietician in a governing body, on the level of the emirate, highlighted that this dissonance is further fostered with the inconsistency between the school and the home environments. Home nurturers, be it parents or otherwise, have great impact on the students' attitudes and behaviors. Their lack of cooperation regarding SNPs leads to uncalled-for confusion among students.

11ESC: “…what is the point of having the child eating healthy in school, and then going home and eat junk food and unhealthy snacks…”

According to a school nurse, who is significantly involved in the National Obesity Prevention Program, one of the most prominent challenges facing the SNPs in Dubai is the failure to engage parents in the implementation of the SNP program which may lead to resistance to change and to limited cooperation with the school administration in applying what is learned by their children about healthy nutrition.

14ESC: “…while we have developed a special system for obese students, parents seem to have an active role in students' lack of commitment to the recommended diet…”

### Theme 4: Need for better performance measurement and oversight

Stakeholders expressed opinion on the need to strengthen the monitoring and evaluation framework for SNPs in Dubai. The quote below highlights an issue not only with the lack of a clear measurement framework, but also with the presence of a dynamic follow up process and accountability framework.

1EM: “when we come to evaluation there is no evidence that says that schools are following the guidelines, there are no violations if there is any kind of violations, and how such a project has impacted the diet behaviors of the students we do not have anything in relation to that…”

A key policymaker in a federal governing body highlighted that there seems to be no clear system for monitoring and evaluating the effectiveness of SNPs.

4EM: “…there is no evidence that proves that the schools are following the guidelines, or any sort of explanation of how such a project has impacted the attitudes and/or behaviors of the students. There are also no consequences if a violation takes place…”

Another informant also highlighted the absence of a concrete accountability framework:

5EC: “…our colleagues in Ministry of Education keep telling us that all is on track on their end. Reality does not show so- not all the food offerings in schools are healthy. This is evident in the reports provided to us by the school nurses…”

### Theme 5: Limited accommodation for health needs and cultural preferences

A stakeholder in the school health section of a governing body emphasized:

5EC: “…sometimes healthy food is available but more expensive than the unhealthy food…”

Moreover, according to a manager who is concerned with the health and wellbeing of students in private schools, there are people from more than 200 nationalities who are co-existing in Dubai. Correspondingly, the school children in this city are quite diverse. It is very challenging to come-up with a selection of healthy food offerings that meets the expectations and preferences of everyone.

21ESM: “…when you look at food habits, a lot goes back to the students' cultural backgrounds…one size will certainly not fit all- giving an Indian food menu might work for a school that is dominated by Indians, but will certainly not work for international western schools or with schools that have mostly Emirati students…”

In addition, some students suffer from chronic morbidities that require special diets. The schools need to cater to their special needs which is not always possible due to limited resources and expertise in the subject matter, as highlighted by a manager at a governmental entity, overseeing the nursing and clinical support services:

6ESC: “…some students are phenylketonuria patients, and others are intolerant to gluten and/or suffer from celiac disease. Those students who have special dietary needs do not know what to do at school. They do not get food offerings that match their health needs…”

According to a senior dietician in a governing body, students are “picky” in their very nature. The challenge is not only to provide healthy food, but also to provide it in a way that is attractive enough for the students. Most school students nowadays complain about the lack of diversity in the type and quality of food items offered.

10ESC: “…most students complain about the lack of food that they prefer in the canteen…students, even those who are aware about healthy choices, are still more attracted to unhealthy food…”

## Discussion

This study revealed that there are five main challenges concerning the planning, execution, and monitoring and evaluation of SNPs in Dubai. These challenges include limited coordination across governing entities, sub-optimal resourcing of SNPs, weakly coordinated implementation, need for better performance measurement and oversight, and limited accommodation for health needs and cultural preferences. These challenges are not unique to the UAE. Several studies have reported similar hurdles in implementing SNPs in developing countries (14, 24–28). Along the same line, it was highlighted that the lack of funding, awareness, and coordination and cooperation among the stakeholders, learning and development opportunities, and enforcement of policies and procedures constitute the main barriers to implementing the school wellness policies in the United States and adopting nutrition guidelines in schools in Canada (29).

On a governance level, the stakeholders in this study highlighted the redundancies and confusion that is resulting from the multiplicity of governing entities involved in setting and maintaining the groundwork of SNPs in Dubai. The evident diffusion of responsibilities, due to the multiplicity of guidelines, was thoroughly reflected upon by almost all the participating stakeholders. Not knowing exactly who is involved, and what are the roles and responsibilities of all involved parties is preventing the formation of effective interdependencies and leading to many inefficiencies. The evident lack of collaboration and limited communication across the differing involved stakeholders may also be due to the lack of clarity in terms of who is doing what (30–32). In response to similar challenges in Nepal, the collaboration between all the involved governing bodies was strengthened through the introduction of strong leadership that is concerned primarily with enhancing the health and wellbeing of school students. Meetings were regularly held to maintain the common ground through discussing program activities, and achievements and challenges. Stakeholders from all tiers were required to coordinate their efforts and collaborate in implementing and expanding the program nationwide (33). Similar challenges, on a governance level, were also evident in developed countries such as the United States of America, where the school food environment seems to be governed by a patchwork of federal, state, and local laws and policies. Accordingly, it was decided for the federal government to maintain primary authority over the school meal programs, and was tasked with consolidating and updating regulations governing the food and nutrient requirements for meals sold or served through the National School Lunch and School Breakfast Programs (34). In order to circumvent the challenge, around governance of SNPs in Dubai, all the steering efforts need to be centralized, through the development of either an independent entity tasked with overseeing the health and wellbeing of students across all schools in Dubai, or a joint entity which enables effective and efficient collaborations, where the whole becomes more than the sum of its parts.

The lack of consolidation on a governance level is expectedly leading to suboptimal resourcing of SNPs. This includes inconsistency of the quality of food offerings, and the limited human and financial capital, as clearly reflected upon by the study participants. Underlying this gap in resourcing is the absence of holistic planning, and of overarching policies and procedures. To address similar gaps in the implementation efforts of SNPs in Nigeria, the Federal Government launched in 2005 the Home-Grown School Feeding and Health Program under the leadership of the Federal Ministry of Education (31, 35). The goal of this program was to provide standardized nutritious meals during the school day to all public and private primary and secondary educational institutions across the country. This national program framework was inaugurated on a state and local levels, with a state steering committee led by the Ministry of Education (35). The need for high-level direction, and for consolidated resourcing including the infrastructure, human and financial capital, and administrative systems to support SNPs implementation is established in the literature (36). Regarding human capital, each school in Dubai would need to identify competent resources, within their respective communities or as new hires, and ensure those resources dedicate enough time to effectively run SNPs. They will also need to instill rigorous learning and development opportunities for the identified human resources to ensure that they are aware of all the requirements and are fully equipped with what it takes to ensure effective planning, implementation, and monitoring and evaluation of SNPs at the level of the respective schools. These learning and development opportunities can be governed and offered by the same entity that will be tasked with overseeing the SNPs in Dubai. In addition, this entity can provide subsidies to support schools with hiring, and/or training those school nutrition officers (37).

The stakeholders also referred to the problematic segregation in implementation efforts of SNPs in Dubai. A unified and standardized implementation plan needs to be developed and implemented in all schools. Along the same lines, the Ministry of Education in Saudi Arabia developed and endorsed the national level “Regulations of Health Conditions for School Canteens” in 2004, and has been regularly updating it since then (38, 39). Such overarching policies has been reported upon in the Kingdom of Saudi Arabia, where they are considered the responsibilities of the General Administration of School Health (40). These policies could help in creating healthy school environments by providing healthy food choices (41). The plan of SNPs in Dubai needs to place students, and their families, at the center and needs to cross the boundaries of schools all the way to the students' households to enhance the chances of success. Gillies et al. (42) highlighted that parent engagement, through communication with their children, is an important way to extend the SNPs beyond the school environment.

The stakeholders also emphasized the need for better measurement of SNPs performance. There are many indicators that are set by differing entities with the intention of evaluating performance. These indicators are not effectively communicated to the stakeholders, and when they are, it does not seem to be clear to the stakeholders how these indicators come together to create an accountability scheme, and to systematically monitor and evaluate the performance of SNPs in Dubai. Similarly, absence of effective monitoring of school store foods, lack of awareness of the policy, and profit-seeking attitudes and behaviors were identified as reasons for ineffective implementation of the such policies in schools of South Korea (43). Hence, the unified plan of SNPs in Dubai that will be set by the overseeing governance entity will need to be supported by an overarching Monitoring & Evaluation framework, composed of a standard set of Key Performance Indicators to measure hard outcomes (e.g., Body Mass Index) and experiences (e.g., satisfaction with implementation and suggestions for improvement). Any observed gap in performance needs to be addressed in a timely manner, and these evidence-driven action plans need to be effectively communicated to the community-at-large for the students and their families to see how the SNPs are continuously improving to better address their needs. For example, Cullen and Watson (41) assessed the statewide impact of the 2004 Texas Public School Nutrition Policy on foods and beverages served or sold in schools. They found that the respective policy changes have improved foods served or sold to students (36).

The stakeholders explained how the current SNPs framework does not adequately accommodate for the health needs and cultural preferences of all the school enrollees in Dubai. Some students are left out because they are unable to afford the high pricing of healthy food offerings. An opportunity exists to explore the role of differing pricing strategies and food subsidies, which may require partnerships with local food providers. This approach will also support the realization of efficiencies, by employing economies of scale, through the bulk purchasing of food items. Moreover, the current offerings do not extensively cover all the diverse preferences of the students residing in Dubai. Hence, we recommend for the SNPs in Dubai policies to be reflective of the spirit of Dubai, inclusive of all ethnicities, with more culturally sensitive programming and implementation, channeling particular attention to the nutritional needs of students with dietary restrictions. This policy needs to include sections that will call for equitable allocation of resources and the standardization of quality of food. It will further necessitate better coordination between the ministries of health and prevention and education.

Accordingly, efforts need to be directed toward unifying the governance structure, the strategic and operational plan, and the monitoring and evaluation framework. This governance structure needs to be characterized by strong leadership, unified standards, and a comprehensive and firmly enacted policy. This is expected to significantly enhance the collaboration and in turn co-creation on the level of Dubai. Through consolidation, processes will become more effective and efficient, where economies-of-scale can be realized. This can be complemented by capacity building of all involved stakeholders which will empower them and maximize their engagement.

To the best of the authors' knowledge, the current study constitutes the first attempt to systematically develop a systemic understanding of SNPs in Dubai, UAE. The inductive, exploratory approach to research adapted for this study enabled the development of in-depth insights about the subject matter. It is worth conducting a follow-up deductive study to confirm the identified findings. This study is also characterized by a few limitations. To start with, the generalizability of the results is limited since the informants who were invited to participate are ones that are either directly or indirectly involved in the implementation of SNPs in Dubai. There is a potentiality for social desirability bias, where the participants might have chosen to respond in a manner that is favorable by others. Moreover, as previously highlighted by Gillies et al. (42), it is crucial to develop a thorough understanding of student perceptions of the policy and of the school food environment. In this study, school students and their parents were not included among the participants; their perspective is important to informing customer-centric policy reformations. Hence, it is recommended for future studies to capture qualitative data of the perspectives of the ultimate receivers: the students and their families. It will also be useful to triangulate qualitative and quantitative data to understand how the various schools are performing, in terms of SNPs, and where the gaps actually are.

## Conclusion

The current study emphasizes that all the involved stakeholders, from the various institutions, need to better collaborate and in turn co-create. This will require the mindful formation of a unified governing body, which develops a single stream of resources, and sets in place a reliable, all-encapsulating and equitable implementation plan along with an overarching monitoring and evaluation framework.

## Data availability statement

The original contributions presented in the study are included in the article/supplementary material, further inquiries can be directed to the corresponding author.

## Ethics statement

The studies involving human participants were reviewed and approved by ethical approval for the study was obtained from the International Review Board (IRB) at the Mohammed Bin Rashid University of Medicine and Health Sciences (MBRU), Dubai, UAE, under the reference number MBRU-IRB-2018-029. The patients/participants provided their written informed consent to participate in this study.

## Author contributions

RA and MA conceptualized the study, supervised the data collection and analysis activities, and contributed to preparing the first draft of the manuscript. NA carried out the data collection activities and contributed to the analysis of data and to the write-up of the first draft of the manuscript. FO contributed to the analysis of data and to the write-up of the manuscript. All authors contributed to the article and approved the submitted version.

## Funding

This study was funded by MBRU College of Medicine internal research grants.

## Conflict of interest

The authors declare that the research was conducted in the absence of any commercial or financial relationships that could be construed as a potential conflict of interest.

## Publisher's note

All claims expressed in this article are solely those of the authors and do not necessarily represent those of their affiliated organizations, or those of the publisher, the editors and the reviewers. Any product that may be evaluated in this article, or claim that may be made by its manufacturer, is not guaranteed or endorsed by the publisher.

## Abbreviations

SNPs: School Nutrition Programs
UAE: United Arab Emirates
MOHAP: Ministry of Health and Prevention
DHA: Dubai Health Authority
MOE: Ministry of Education
KHDA: Knowledge and Human Development Authority of Dubai
IRB: International Review Board
MBRU: Mohammed Bin Rashid University of Medicine and Health Sciences.
